# Poisoned by Tobacco Juice

**Published:** 1865-01

**Authors:** 


Poisoned by Tobacco Juice.—A young man, named Richard Edmondson, a cotton piercer at Messrs. Garnett and Horsfall's, Low Moor, near Clitheroe, died last week somewhat suddenly, with all the symptoms of having been poisoned. His pulse was quick and feeble, his eyes dilated and insensible to light; the heart was perfectly paralyzed, his muscles rigid, and he was unable to swallow. This was his condition before death. The coroner ordered a *post-mortem*

examination of the body to be made by Dr. Scott, of Clitheroe. He found the vessels of the brain swollen and filled with black blood, together with extravasation of blood in the ventricles of the brain. "These appearances," he deposed, "led me to conclude that the deceased had taken some narcotic poison. The deceased was very much emaciated. After hearing all the evidence I attribute the cause of his death to the chewing of Limerick roll tobacco and his having swallowed the juice. It is a kind of poison that acts on the brain, and is an irritant and compound poison. It is not used in medicine now. I should not like to give a person thirty grains of the unprepared tobacco. Tobacco gains power according to the way in which it is manufactured, and the Limerick roll is exceedingly strong tobacco." The coroner summed up, and the jury returned a verdict "That the deceased died from the effect of having chewed Limerick roll tobacco and swallowing the juice thereof, which has acted upon the stomach as a narcotic poison."—*The Chemist and Druggist.*

